# Alcohol and drug use among clients receiving internet-delivered cognitive behavior therapy for anxiety and depression in a routine care clinic – Demographics, use patterns, and prediction of treatment completion and outcomes

**DOI:** 10.1016/j.invent.2021.100490

**Published:** 2021-12-14

**Authors:** Christopher Sundström, Michael Edmonds, Joelle N. Soucy, Nickolai Titov, Blake F. Dear, Heather D. Hadjistavropoulos

**Affiliations:** a Department of Psychology, University of Regina, 3737 Wascana Parkway, Regina, SK S4S 0A2, Canada; b Centre for Psychiatry Research, Department of Clinical Neuroscience, Karolinska Institutet, & Stockholm Health Care Services, Region Stockholm, Norra Stationsgatan 69, 113 64, Stockholm, Sweden; c Department of Psychology, Stockholm University, 106 91 Stockholm, Sweden; d eCentreClinic, Department of Psychology, Macquarie University, Sydney, NSW 2109, Australia

**Keywords:** OTU, Online Therapy Unit, Internet-delivered cognitive behavior therapy, Depression, Anxiety, Alcohol, Drugs, Substance use

## Abstract

**Background:**

Research shows that alcohol and drug use among mental health clients is common and has the potential to negatively impact treatment outcomes. Internet-delivered cognitive behavior therapy (ICBT) as a treatment for anxiety and depression is on the rise, but little is known about the prevalence of alcohol and drug use among clients and how this use affects treatment completion and outcomes.

**Objective:**

The objective of the current study was to explore the prevalence of alcohol and drug use among clients in ICBT for depression and anxiety, and to investigate the impact of alcohol and drug use on treatment completion and symptom outcomes.

**Material and methods:**

Data was collected from 1155 clients who participated in two randomized ICBT trials for depression and anxiety, conducted in a routine care clinic. Thirty-five individuals reporting severe substance use when applying to the trials were excluded. Demographic variables, and alcohol and drug use were measured at screening, and measures of depression and anxiety were administered at pre- and post-treatment.

**Results:**

Four out of five clients reported having used alcohol in the past year, while one in five reported having used drugs in the past year. Around a third of clients had reported either problematic alcohol use, drug problems, or both. The analyses showed that drug problems, and combined alcohol and drug problems were negatively associated with treatment completion, but neither alcohol nor drug use had an impact on depression and anxiety outcomes.

**Conclusions:**

Alcohol and drug problems are likely to be present among a large proportion of patients using ICBT for anxiety and depression. This may not be a barrier to treatment benefit, at least when those with severe alcohol and drug problems have been excluded.

## Highlights

• Alcohol and drug use are known to be common among psychiatry patients.
• We investigated alcohol and drug use among clients receiving internet-delivered CBT.
• Around a third of clients had an alcohol and/or drug use problem.
• These problems did not seem to negatively affect depression and anxiety outcomes.

## 1 Introduction

Epidemiological studies conducted in the general population over the past three decades provide unequivocal evidence that substance use disorders (SUD) and mood and anxiety disorders commonly co-occur (Lai et al., 2015), with an estimated 20% of those with a mood disorder and 15% of those with an anxiety disorder also meeting criteria for a SUD (Grant et al., 2004). Studies conducted in patient samples confirm this comorbidity is at least equally high in mental health clinics, with alcohol use disorder being the most common SUD, followed by cannabis use disorder (Hunt et al., 2016; Margolese et al., 2004; Toftdahl et al., 2016). The link between mood and anxiety disorders and SUD is complex and there are differing explanations for this comorbidity (Castillo-Carniglia et al., 2019). Self-medication remains the most popular hypothesis (Abraham and Fava, 1999; Robinson et al., 2011; Turner et al., 2018), although studies indicate both mood and anxiety disorders can sometimes be substance-induced (Kenneson et al., 2013; Kushner et al., 2000). Regardless of etiology, it is important for mental health clinics to appropriately assess substance use among patients. SUD should ideally be treated concurrently with mood and anxiety disorders, as focusing solely on one of the two seems less effective (Kelly et al., 2012). Further, milder forms of substance use (e.g., exceeding national drinking guidelines, weekend binge drinking, frequent cannabis use) are also problematic in clinical settings. For example, studies show that hazardous alcohol use and regular cannabis use are associated with poorer recovery among psychiatric patients (Bahorik et al., 2016; Bahorik et al., 2017; Bricker et al., 2007). Substance use can interact negatively with psychopharmacological treatment (Worthington et al., 1996) and may directly interfere with psychological treatment, for instance when alcohol is used as a safety behavior among people with social anxiety disorder (Morris et al., 2005) or when cannabis is used for emotion regulation of posttraumatic stress (Bonn-Miller et al., 2011). Importantly, suicidal ideation and suicide are particularly common among patients with combined substance use problems and psychiatric problems (Davis et al., 2008; Yuodelis-Flores and Ries, 2015). Still, research suggests that substance use is rarely given much clinical attention in outpatient psychiatry (Satre et al., 2014), with women less likely to have substance use addressed by clinicians (Weisner and Matzger, 2003).

Internet-delivered cognitive behavior therapy (ICBT) is an increasingly common treatment modality for people with mental health problems, usually consisting of modules based on cognitive behavior therapy delivered online, sometimes with a therapist guiding the patient through the program (Andersson, 2018). Many studies on ICBT for depression and anxiety have been conducted over the past 20 years, showing significant effects in the treatment of anxiety and depression (Karyotaki et al., 2017; Olthuis et al., 2016), results that have been replicated in ICBT clinics (Etzelmueller et al., 2020). In Canada, the publicly-funded Online Therapy Unit (OTU) is situated in the province of Saskatchewan and has been offering free-of-cost ICBT for depression and anxiety to its residents since 2010. Saskatchewan has one of the highest alcohol consumption rates of all provinces in Canada (Canadian Centre on Substance Use and Addiction, 2019) and clients are excluded from ICBT for depression and anxiety if alcohol and/or drug use is deemed to be the primary source of concern or severe in nature; nevertheless many clients still do experience hazardous and sometimes harmful use of alcohol or drugs and little is known about how these clients respond to ICBT.

There is growing interest in offering ICBT for depression and anxiety in Canada (Health Quality Ontario, 2019) as well as other countries (Titov et al., 2018; Titov et al., 2019). As alcohol and drug use may interfere with psychiatric treatment, examining the prevalence of alcohol and drug use problems among people using these emerging services, and investigating whether these problems affect clinical outcomes is vital to understand who is likely to benefit from these services, and how they fit within the health system. However, despite the many randomized trials conducted on ICBT for anxiety and depression over the past 20 years, research on alcohol and drug use among these clients is sparse. To our knowledge, there is only one previously published study investigating both alcohol and drug use in ICBT for anxiety and depression. This study found that hazardous alcohol and drug use in a routine care ICBT clinic was associated with worse outcomes for patients with social anxiety disorder and panic disorder, but not for patients with depression (Gajecki et al., 2014). The current study similarly investigates whether alcohol and drug use are associated with treatment completion and clinical outcomes in ICBT for depression and anxiety, with the goal of adding to the literature by presenting a detailed description of alcohol and drug use patterns among ICBT clients.

## 2 Material and methods

### 2.1 Design and ethics

This study used data from two randomized controlled trials conducted at the Online Therapy Unit (OTU; www.onlinetherapyuser.ca) where the *Wellbeing Course*, a transdiagnostic online program for people with anxiety and depression, was evaluated (for details on the course, see Titov et al., 2015). The trials received ethics board approval from the University of Regina, were registered (ClinicalTrials.gov Identifier: NCT03304392; NCT03684434), and conducted between 2017 and 2019. Details on the design of the trials are available elsewhere (Hadjistavropoulos et al., 2020; Soucy et al., 2021). In brief, the first trial (Hadjistavropoulos et al., 2020) compared the benefits of therapists providing support once-weekly versus therapists providing support once-weekly supplemented with a one-business-day response to all client emails with no significant differences found in treatment completion or outcomes (Hadjistavropoulos et al., 2020); whereas the second (Soucy et al., 2021) compared the benefits of clients receiving ICBT with and without a preceding online motivational interviewing lesson and similarly found no significant group differences in treatment completion or outcomes (Soucy et al., 2021). As no differences were found between experimental groups in these two trials and both trials involved the same treatment program, data was combined to maximize power in the current analysis.

### 2.2 Participants and procedure

Clients included in the two trials were mostly referred from healthcare providers who instructed them to visit the OTU website, where information about the *Wellbeing Course* and an online screening assessment was provided. The survey covered contact details (e.g., telephone number, email address), demographic information (e.g., sex, ethnicity, location etc.), information about depression and anxiety, alcohol and drug use, and relevant background information (e.g., medical history, mental health history, symptoms). Upon completing the survey, a telephone enrollment call with unit staff was booked through an online appointment booking software. In the telephone enrollment call, potential clients were asked a series of follow-up questions to the screening questionnaire by unit staff to ensure eligibility and were once again asked to consent to participate. To be included in either of the trials, clients had to: (a) be 18 years or older; (b) be a Saskatchewan resident; (c) have access to the internet; and (d) self-report problems with depression, defined as a score of ≥5 on the Patient Health Questionnaire 9-item scale (PHQ-9) (Kroenke et al., 2001), and/or problems with anxiety, defined as a score ≥ 5 on the Generalized Anxiety Disorder 7-item scale (GAD-7) (Spitzer et al., 2006). Clients were excluded from the trial if they presented with: (a) risk of suicide; (b) other severe mental health or medical conditions (e.g. unmanaged bipolar disorder, schizophrenia etc.); (c) severe alcohol or substance use problems as assessed by the interviewer in the telephone enrollment call; (d) low motivation to engage with online treatment as assessed by the interviewer in the telephone enrollment call; (e) ongoing or impending significant mental health treatment, defined as seeing a mental health professional more frequently than twice a month; or (f) hospitalization for mental health reasons within the past year. After consent had been collected, clients were granted access to the *Wellbeing Course* through a secure website. All clients were guided by therapists, who contacted them at least weekly over the 8-week treatment period.

### 2.3 Measures

#### 2.3.1 Alcohol and drug use

Alcohol use was measured with the Alcohol Use Disorder Identification Test (AUDIT), which has demonstrated good psychometric properties in numerous studies over the past 25 years (Reinert and Allen, 2007). The instrument consists of ten items scored between 0 and 4, with a total score ranging from 0 to 40. The first three items of the AUDIT assess alcohol consumption patterns: question 1 asks how often the participant drinks alcohol (with those responding “never” being directed to question 9), question 2 asks how many drinks the participants usually consume when drinking, and question 3 asks about frequency of heavy episodic drinking (i.e. having 6 drinks or more on one occasion). Items 4–6 assess dependence (i.e. impaired control over drinking, increased salience of drinking, morning drinking) and items 7–10 assess alcohol-related harm (i.e. guilt after drinking, blackouts, injuries, concern from others) (Higgins-Biddle and Babor, 2018). A sum score of 8–14 for men and 6–14 for women on the AUDIT indicates hazardous use, while a sum score of 15–19 indicates harmful use and a sum score of ≥20 indicates probable dependence (Saunders et al., 1993). The first three items about consumption can also be summarized into AUDIT-C (Bradley et al., 2007; Meneses-Gaya et al., 2010), with recommended cut-offs for hazardous drinking at ≥5 for men and ≥4 for women, when studied in a psychiatric context (Dawson et al., 2005) and among online help-seekers (Khadjesari et al., 2017).

To assess drug use, clients were administered the Drug Use Disorder Identification Test (DUDIT) (Berman et al., 2005). Several studies have shown DUDIT to have good to excellent internal consistency (0.74–0.97) as well as favorable sensitivity and specificity (Hildebrand, 2015). This questionnaire has eleven items scored between 0 and 4, with a total score ranging from 0 to 44. The first four items assess drug use patterns: question 1 asks how often the participant uses other substances than alcohol (with those responding “never” being directed to question 10), question 2 asks whether the participant usually uses more than one drug when using, question 3 asks about how many times the participant takes drugs on a typical day of using, and question 4 asks how often drugs heavily influence them. Items 5–8 assess dependence symptoms (i.e. craving, impaired control over using, increased salience of using, morning using) and items 9–11 drug-related harm (i.e. guilt after using, injuries, concern from others). A sum score of 2–24 for women and 6–24 for men on the DUDIT has been suggested as indicative of drug problems and a sum score of ≥25 as indicative of drug dependence (Berman et al., 2005).

#### 2.3.2 Depression and anxiety

The current study made use of the following measures administered at pre-treatment and 8-weeks post-treatment. Notably, all participants had to complete all items of symptom questionnaires for participation in treatment, meaning missing data at the item level is not a concern in this dataset.

Depression was measured with the Patient Health Questionnaire (PHQ-9), a self-report questionnaire with nine items screening for depression symptoms over the past two weeks. Items are rated 0 to 3, with total scores ranging from 0 to 27 (Kroenke et al., 2001),. The instrument has good psychometric properties (Kroenke et al., 2010). One item in the PHQ-9 addresses suicidal ideation.

Generalized anxiety was measured with the Generalized Anxiety Disorder 7-item Scale (GAD-7), a self-report questionnaire with seven items screening for anxiety symptoms over the past two weeks (Spitzer et al., 2006). Items are rated 0 to 3 with total scores ranging from 0 to 21. The GAD-7 has strong psychometric properties (Spitzer et al., 2006).

Social anxiety was measured with the Social Interaction Anxiety 6-item Scale and the Social Phobia 6-item Scale (SIAS-6/SPS-6) which have both been found to be psychometrically sound measure of social anxiety when measured together or separately (Peters et al., 2012). Participants respond to each item on a 0 to 4 scale with items being summed to yield a total score between 0 and 48.

Panic disorder symptoms were measured with Panic Disorder Symptom Severity Scale (PDSS). This self-report questionnaire has seven items that ask about common symptoms of panic disorder on a scale from 0 to 4 scale. The PDSS has been found to be psychometrically sound, including good test-retest reliability and high internal consistency (Houck et al., 2002).

#### 2.3.3 Course completion

Course completion was measured by whether each client had accessed all five lessons in the *Wellbeing Course.*

### 2.4 Statistical analysis

Descriptive statistics were used to summarize demographic and clinical characteristics among all clients, among clients without any alcohol or drug use problems, among clients with hazardous/harmful alcohol use, among clients with drug use problems, and among clients who reported both problematic hazardous/harmful alcohol use and drug problems. A series of one-way analysis of variance (ANOVA) calculations were conducted to investigate whether there were any differences among the groups in terms of age and severity of depression and anxiety, using the Tukey-Kramer post-hoc test to control for mass significance. Chi square tests were also used to explore differences among the groups in terms of categorical demographics such as gender, marital status and income. To provide a detailed picture of alcohol and drug use patterns among clients, descriptive statistics were used to investigate endorsement of individual items on the AUDIT and DUDIT. Previous research has found differences in drinking patterns among men and women in psychiatry (Satre et al., 2011), and for this reason, we used chi-square to investigate endorsement of items also by gender. Next, we conducted logistic regressions to investigate whether AUDIT and DUDIT scores predicted treatment completion and used chi-square to investigate whether treatment completion differed among the five substance use categories (i.e. no problematic substance use, hazardous alcohol use, harmful alcohol use, drug problems, combined alcohol and drug problems). Several multiple linear regression analyses were then conducted to investigate whether AUDIT and DUDIT scores predicted change in depression and suicidal ideation, generalized anxiety, social anxiety, and panic disorder symptoms over the 8-week course of treatment. A change score representing the difference between scores on each instrument measuring depression or anxiety at pre- and post-treatment was entered as the dependent variable and the client's initial scores on the instrument were entered in the first block. In the second block, AUDIT and DUDIT scores provided by clients at screening were entered stepwise as potential predictor variables in addition to gender and age. We also used one-way ANOVAs to compare differences in anxiety and depression change scores between the groups. In a secondary analysis, missing data was imputed using last-observation-carried-forward (LOCF). As the study was seen as mainly exploratory, it was decided that no corrections for multiple testing would be applied.

## 3 Results

### 3.1 Demographic and clinical characteristics

Out of 1190 individuals, thirty-five (2.9%) were excluded due to primary alcohol and/or substance use problems. Thus, in all, 1155 clients were included in the two trials and subsequently included in the current analysis. Demographic and clinical characteristics of the present sample are presented in Table 1.

**Table 1:** Demographic and clinical characteristics of the sample.

	All clients	No problematic use	Problematic alcohol use only		Drug use problems only	Combined alcohol and drug problems	Chi^2^/*F*	*p*
			Hazardous	Harmful				
N (%)	1155 (100)	798 (68.4)	136 (11.8)	24 (2.1)	126 (10.9)	71 (6.1)		
Age, mean (SD)	37.0 (13.0)	38.7 (13.5)	36.0 (11.3)	36.6 (11.1)	31.7 (9.9)	30.0 (9.8)	14.502	<0.001⁎⁎
Female, n (%)	871 (75.4)	594 (74.4)	99 (72.8)	16 (66.7)	100 (79.4)	62 (87.3)	8.397	0.078
White, n (%)	1043 (90.3)	724 (90.7)	124 (91.2)	22 (91.7)	110 (87.3)	63 (88.7)	1.829	0.767
Urban n (%)	475 (41.1)	305 (38.2)	61 (44.9)	11 (45.8)	60 (47.6)	38 (53.5)	10.481	0.033⁎
Married/living with partner n (%)	512 (62.5)	519 (65)	88 (64.7)	13 (54.2)	67 (53.2)	35 (49.3)	13.144	0.011⁎
University degree n (%)	400 (34.6)	294 (36.8)	51 (37.5)	10 (41.7)	25 (19.8)	20 (28.2)	16.227	0.003⁎⁎
Employed n (%)	852 (73.9)	578 (72.5)	108 (79.4)	21 (87.5)	88 (70.4)	57 (80.3)	7.520	0.111
AUDIT mean (SD) (range)	3.69 (3.81) (0–20)	2.01 (1.72) (0–7)	8.89 (2.31) (6–14)	16.67 (1.46) (15–20)	2.90 (1.81) (0–7)	9.65 (3.00) (6–18)	868.166	<0.001⁎⁎
AUDIT-C mean (SD) (range)	2.49 (2.14) (2–13)	1.73 (1.52) (2–8)	5.21 (1.89) (2–13)	7.13 (1.73) (5–11)	2.11 (1.51) (2–7)	4.90 (1.73) (2–10)	240.574	<0.001⁎⁎
DUDIT mean (SD) (min–max)	1.30 (3.34) (0–26)	0.13 (0.67) (0–5)	0.35 (1.10) (0–5)	0.83 (1.49) (0–5)	6.63 (4.91) (2–23)	6.99 (5.56) (2–26)	355.160	<0.001⁎⁎
GAD-7, mean (SD)	12.50 (5.02)	12.11 (5.16)	13.05 (4.30)	12.71 (4.55)	13.49 (4.92)	13.96 (4.46)	4.387	0.002⁎⁎
PHQ-9, mean (SD)	13.38 (5.64)	12.86 (5.60)	13.43 (5.75)	14.21 (3.98)	15.16 (5.37)	15.72 (5.64)	8.209	<0.001⁎⁎
PHQ-9, suicide item, mean (SD)	0.24 (0.53)	0.21 (0.491)	0.20 (0.499)	0.39 (0.583)	0.40 (0.725)	0.32 (0.606)	4.433	0.001⁎⁎
Any suicidal ideation, PHQ-9 Item 9, n (%)	216 (19.4)	135 (17.4)	21 (15.8)	8 (34.8)	34 (29.8)	18 (26.1)	29.157	0.004⁎⁎
SIAS-6/SPS-6, mean (SD)	14.79 (11.06)	14.09 (10.74)	14.10 (10.75)	14.19 (18.29)	18.29 (11.71)	18.88 (12.84)	5.281	<0.001⁎⁎
PDSS, mean (SD)	7.55 (5.92)	7.29 (5.90)	7.33 (5.66)	8.26 (5.09)	9.12 (6.13)	8.04 (6.24)	2.637	0.033⁎

Abbreviations: AUDIT = Alcohol Use Disorder Identification Test; AUDIT-C = Alcohol Use Disorder Identification Test – Consumption; DUDIT = Drug Use Disorder Identification Test; GAD-7 = Generalized Anxiety Disorder 7-item Scale; PHQ-9 = Patient Health Questionnaire; SIAS-6 = Social Interaction Anxiety Scale 6-item Scale SPS-6 = Social Phobia 6-item Scale; PDSS = Panic Disorder Symptom Severity Scale.

⁎ ≤0.05.

⁎⁎ ≤0.01.

As for alcohol, 160 clients had a problematic alcohol use (13.9%), with 136 having a hazardous alcohol use (11.8%), and 24 (2.1%) having harmful alcohol use. As for drugs, 126 (10.9%) clients had drug problems. Further, 71 (6.1%) clients had combined alcohol and drug problems. Those with drug problems and combined alcohol and drug problems were significantly younger, less likely to be married, and more likely to live in a large city compared to those with no problematic use. Those with drug problems were significantly less likely to hold a university degree. Further, those with drug problems and combined alcohol and drug problems scored significantly higher on both measures of depression, generalized anxiety, and social anxiety compared to those with no problematic use. Those with drug problems also scored significantly higher on panic disorder symptoms compared to those with no problematic use. Those with harmful alcohol use and those with drug problems had a significantly higher prevalence of suicidal ideation compared to those with no problematic use.

### 3.2 Alcohol use

#### 3.2.1 AUDIT items

To investigate alcohol use patterns among clients in the sample, we first examined individual AUDIT items that were rated as ≥1 (i.e. endorsement). In the sample, 78.4% (*n* = 905) endorsed item 1 (having consumed alcohol in the past year), with no significant differences between men and women (*p* = 0.807; see Table 2).

**Table 2:** Alcohol Use Disorder Identification Test – item endorsement (scoring ≥1)^a^.

	All	% endorsed		Chi^2^	*p*
		Female	Male		
Alcohol consumption					
1. Frequency of drinking: How often do you have a drink containing alcohol? (above never)	905 (78.4)	681 (78.2)	224 (78.9)	0.060	0.807
2. Typical quantity: How many drinks containing alcohol do you have on a typical day when you are drinking? (3 or more) (above 1 or 2)	285 (31.5)	201 (29.5)	84 (37.5)	4.981	0.026⁎
3. Frequency of heavy drinking: How often do you have six or more drinks on one occasion? (above never)	514 (56.8)	366 (53.7)	148 (66.1)	10.438	0.001⁎⁎

Symptoms of dependence					
4. Impaired control over drinking: How often during the last year have you found that you were not able to stop drinking once you had started? (above never)	141 (15.6)	92 (13.5)	49 (21.9)	8.968	0.003⁎⁎
5. Increased salience of drinking: How often during the last year have you failed to do what was normally expected from you because of drinking? (above never)	107 (11.8)	76 (11.2)	31 (13.8)	1.161	0.281
6. Morning drinking: How often during the last year have you needed a first drink in the morning to get yourself going after a heavy drinking session? (above never)	15 (1.7)	13 (1.9)	2 (0.9)	1.068	0.301

Alcohol-related problems/harms					
7. Guilt after drinking: How often during the last year have you had a feeling of guilt or remorse after drinking? (above never)	275 (30.4)	196 (28.8)	79 (35.3)	3.353	0.067
8. Blackouts: How often during the last year have you been unable to remember what happened the night before because you had been drinking? (above never)	160 (17.7)	118 (17.3)	42 (18.8)	0.234	0.628
9. Alcohol-related injuries: Have you or someone else been injured as a result of your drinking? (above no)	74 (6.4)	46 (5.3)	28 (9.9)	7.485	0.006⁎⁎
10. Others concern about drinking: Has a relative or friend or a doctor or another health worker been concerned about your drinking or suggested you cut down? (above no)	107 (9.3)	68 (7.8)	39 (13.7)	8.945	0.003⁎⁎

⁎ ≤0.05.

⁎⁎ ≤0.01.

a Items 2–8 only administered to those endorsing item 1.

Among those endorsing alcohol consumption in the past year, men were significantly more likely to report having more than 2 drinks on a regular drinking occasion (item 2; *p* = 0.026), and men were also significantly more likely to report heavy episodic drinking (item 3; *p* = 0.001). Item 3 on heavy episodic drinking was also the most frequently endorsed item in the AUDIT, among those endorsing alcohol use (*n* = 514; 56.8%). Men were significantly more likely to report impaired control over drinking (item 4; *p* = 0.003), to report alcohol-related injuries (item 9; *p* = 0.006), and to report concern from others (item 10; *p* = 0.003). The least frequently endorsed item was morning drinking, which was endorsed by fifteen clients in the sample (item 6; *p* = 0.301).

#### 3.2.2 AUDIT sum scores

According to their AUDIT overall scores, 160 (13.9%) clients in the sample met criteria for hazardous or harmful alcohol use (without drug problems); 136 (85%) clients had a hazardous use, and 24 clients (15%) had harmful alcohol use.

### 3.3 Drug use

#### 3.3.1 DUDIT items

Similar to the AUDIT, we investigated endorsement of individual items on the DUDIT. In the sample, 19.4% (*n* = 224; see Table 3) reported having used drugs in the past year, with men significantly more likely to report such use (item 1; *p* ≤ 0.001).

**Table 3:** Drug Use Disorder Identification Test – item endorsement (scoring ≥1)^a^.

	All	% endorsed		Chi^2^	*p*
		Female	Male		
Drug consumption					
1. Frequency of use: How often do you use drugs other than alcohol?⁎ (above never)	224 (19.4)	148 (17.0)	76 (26.8)	13.073	<0.001⁎⁎
2. Typical quantity: Do you use more than one type of drug on the same occasion? (above never)	20 (8.9)	13 (8.8)	7 (9.2)	0.11	0.916
3. Frequency of use per occasion: How many times do you take drugs on a typical day when you use drugs? (above 0)	212 (94.6)	138 (93.2)	74 (97.4)	1.685	0.194
4. Frequency of heavy use: How often are you influenced heavily by drugs? (above never)	97 (43.3)	64 (43.2)	33 (43.4)	0.001	0.980

Symptoms of dependence					
5. Craving: Over the past year, have you felt that your longing for drugs was so strong that you could not resist it? (above never)	42 (18.8)	25 (16.9)	17 (22.4)	0.989	0.320
6. Impaired control over using: Has it happened, over the past year, that you have not been able to stop taking drugs once you started? (above never)	23 (10.3)	15 (10.1)	8 (10.5)	0.008	0.927
7. Increased salience of using: How often over the past year have you taken drugs and then neglected to do something you should have done? (above never)	65 (29.0)	41 (27.7)	24 (31.6)	0.366	0.545
8. Morning using: How often over the past year have you needed to take a drug the morning after heavy drug use the day before? (above never)	8 (3.6)	5 (3.4)	3 (3.9)	0.047	0.828

Drug-related problems/harms					
9. Guilt after using: How often over the past year have you had guilt feelings or a bad conscience because you used drugs? (above never)	73 (32.6)	46 (31.1)	27 (35.5)	0.452	0.502
10. Drug-related injuries: Have you or anyone else been hurt (mentally or physically) because you used drugs? (above no)	36 (3.1)	21 (2.4)	15 (5.3)	5.845	0.016⁎
11. Others concern about using: Has a relative or a friend, a doctor or a nurse, or anyone else, been worried about your drug use or said to you that you should stop using drugs? (above no)	48 (4.2)	25 (2.9)	23 (8.1)	14.698	<0.001⁎⁎

⁎ ≤0.05.

⁎⁎ ≤0.01.

a Items 2–9 only administered to those endorsing item 1.

Frequency of heavy use was the most frequently endorsed item among those reporting use, and there was no significant difference between men and women on this item (item 4; *p* = 0.980).^1^ However, men were significantly more likely to report injuries (item 10; *p* = 0.016) and concern from others (item 11; *p* ≤ 0.001). The least frequently endorsed item was morning use, which was endorsed by eight individuals (item 8; *p* = 0.828).

#### 3.3.2 DUDIT sum scores

According to their DUDIT overall scores, 126 (10.9%) clients in the sample met criteria for having a drug problem (without hazardous alcohol or harmful alcohol use), and one individual had a score of 26 indicating drug dependence.

### 3.4 Combined alcohol and drug problems

In addition to those with a hazardous alcohol use, harmful alcohol use or drug problems, 71 (6.1%) clients met criteria for combined alcohol and drug problems (i.e. having hazardous or harmful alcohol use as well as drug problems).

### 3.5 Alcohol and drug use as predictors of treatment completion

Of the 1155 clients included in the analyses, 1100 logged in and began the *Wellbeing Course*. Of these, 770 (66.7%) completed treatment (i.e. accessed all five lessons; see Table 4). Those with drug problems and combined alcohol and drug problems were significantly less likely to complete the course. Entering total AUDIT and DUDIT scores alongside age and gender as predictor variables into a stepwise logistic regression model predicting program completion revealed that DUDIT scores negatively predicted treatment completion (Exp (B) = 0.957, *p* = 0.03) while age (Exp (B) = 1.034, *p* < 0.001) and being male (Exp (B) = 1.402, *p* = 0.044) positively predicted treatment completion.

**Table 4:** Treatment completion and therapy outcomes among clients in problematic substance use categories.

	All clients	No problematic use	Problematic alcohol use		Drug problems	Combined alcohol and drug problems	Chi 2/*F*	*p*
			Hazardous	Harmful				
Participants at pre-treatment, n (%)	1155	798 (69.1)	136 (11.8)	24 (2.1)	126 (10.9)	71 (6.1)		
Treatment completion, n (%)	770 (66.6)	556 (69.7)	88 (64.7)	15 (62.5)	73 (57.9)	38 (53.5)	13.513	0.009⁎⁎
GAD-7 change score, mean (SD)	−6.24 (4.85)	−6.20 (4.88)	−6.43 (4.80)	−5.50 (3.93)	−6.68 (5.16)	−5.91 (4.50)	0.352	0.843
PHQ-9 change score, mean (SD)	−5.78 (5.16)	−5.77 (5.24)	−5.59 (4.73)	−5.00 (4.16)	−6.77 (5.43)	−4.84 (5.26)	1.180	0.318
PHQ-9, suicide item change score, mean (SD)	−0.104 (0.484)	−0.083 (0.46)	−0.043 (0.506)	−0.222 (0.428)	−0.307 (0.569)	−0.116 (0.544)	4.278	0.002⁎⁎
PHQ-9, any suicidal ideation at post, n (%)	78 (9.51%)	54 (9.2%)	9 (9.6%)	3 (16.7%)	6 (8.0%)	6 (14.0%)	2.344	0.673
PDSS change score, mean (SD)	−3.09 (4.63)	−3.01 (4.63)	−2.84 (4.57)	−3.94 (3.62)	−4.41 (4.95)	−2.05 (4.21)	2.347	0.053
SIAS change score, mean (SD)	−3.31 (6.54)	−3.34 (6.64)	−2.16 (6.25)	−3.00 (5.18)	−4.28 (5.76)	−4.00 (7.43)	1.270	0.280

^⁎^ ≤0.05.

⁎⁎ ≤0.01.

### 3.6 Alcohol and drug use as predictors of symptom change

#### 3.6.1 Depression symptoms

Neither initial scores on AUDIT and DUDIT, age nor gender significantly predicted depression symptom change scores, and there were no significant differences in change scores. There were no significant differences in change scores, but those with drug problems reported a significantly greater reduction in the PHQ-9 suicidal ideation item as compared to those without substance use problems (*p* = 0.002).

#### 3.6.2 Anxiety symptoms

Similarly, neither initial scores on AUDIT and DUDIT, age nor gender were found to significantly predict generalized anxiety symptom change scores, panic disorder symptom change scores, or social anxiety symptom change scores. Further, there were no significant differences in change scores.

#### 3.6.3 Sensitivity analyses

Due to attrition, 31.6% of participants in the course did not complete post-treatment measures. Thus, to examine the potential impact of missing data, all regression analyses were repeated using last-observation-carried-forward approach for missing data. There were no major differences between the outcomes of these analyses compared with those presented above, and hence the results of these analyses are not presented.

## 4 Discussion

This study examined alcohol and drug use among 1155 patients who enrolled with the Online Therapy Unit for treatment of anxiety and depression, and also investigated whether drug and alcohol use were associated with course completion or change in symptoms of depression and anxiety.

### 4.1 The impact of substance use problems on depression and anxiety outcomes

Problematic alcohol use (hazardous and harmful alcohol use) and drug problems were found to be common among these clients, with almost one in three of the sample having either a problematic alcohol use, a drug problem, or both. Those with drug problems and/or combined alcohol and drug problems were younger, more likely to live in a big city, less likely to be married, and less likely to hold a university degree. They also reported greater depression, general anxiety, social anxiety, and panic disorder symptoms as well as greater prevalence of suicidal ideation at intake. Those with drug problems and combined alcohol and drug problems were also significantly less likely to complete treatment, and having a higher DUDIT score at screening negatively predicted treatment completion. In terms of symptom change scores, however, we found no significant differences, apart from the finding that reductions in suicidal ideation over the course of treatment were significantly larger among those with drug problems compared to those with no problematic use.

### 4.2 Substance use patterns

The finding that 80% of clients report alcohol use in the past year corresponds closely with the reported prevalence of alcohol use in the Canadian general population (Canadian Centre on Substance Use and Addiction, 2019). Prevalence of heavy episodic drinking, however, was slightly lower in our sample when compared to a recent survey conducted in the general population in Saskatchewan (the province where the OTU is situated). That is, 28% of men in the general population survey reported heavy episodic drinking at least once a month over the past year, while in our sample this number was 19.7% (56/284). Similarly, 11.8% of females in the Saskatchewan general population reported monthly heavy drinking, compared to 8.8% (77/871) of females in our sample (*Statistics* Canada, 2019). It should be noted, however, that there are limitations to this comparison: heavy drinking in the survey was defined as 4 or more drinks for women and 5 or more drinks for men, while the AUDIT, used in the current study, defines it as 6 or more drinks regardless of gender. There may therefore be a slight underestimation of heavy drinking in our sample. Also, as noted, 35 individuals were excluded when applying for the trials in this study due to severe substance use problems. Nevertheless, results indicate our sample is comparable to the general population when it comes to frequency of alcohol use. This does not seem to be the case with drug use; 19% reported drug use in the past year, which is almost twice as high as the prevalence of past year cannabis use in Saskatchewan in 2012 (10.2%) (Health Canada: Canadian Alcohol and Drug Use Monitoring Survey, 2013). As previously noted, it is rare that individuals reporting use of other drugs than cannabis are accepted into the *Wellbeing Course* at the OTU. Differences in past year drug use among men and women in the general population were similarly twice as high in our sample (7% of women in general population vs. 17% our sample; 13.7% of men in general population vs. 26.8% in our sample) (Health Canada: Canadian Alcohol and Drug Use Monitoring Survey, 2013).

### 4.3 Comparisons to previous research

When comparing our results to the only previously published study investigating problematic alcohol and drug use among clients in ICBT, one similarity is that around a third of clients in both studies reported a substance use problem (30.9% in our study vs 32.4% in Gajecki et al) (Gajecki et al., 2014). Another similarity is that, in both studies, drug problems and combined alcohol and drug problems were associated with lower likelihood of completion of treatment modules. Yet, there are several noticeable differences. Problematic alcohol use in our sample was much lower; 13.9% among clients in our sample met criteria for problematic alcohol use, as compared to 24.1% in the previous study. It remains unclear why this difference emerged, as Canada and Sweden have similar rates of alcohol use in the population (WHO, 2018). Conversely, prevalence of drug problems (10.9%) in our study was more than double the prevalence reported by Gajecki et al. (4.6%). Notably, Gajecki et al. defined drug problems using a DUDIT score of 1 or greater, while the present study defined drug problems using cutoff scores of greater than 6 for men and greater than 2 for women, indicating that the difference in prevalence of drug problems is in fact even larger. The greater prevalence of drug problems in our sample likely reflects that cannabis use is around four times as common in Canada compared to Sweden (UNODC, 2011). Due to the recent legalization, there is also less risk related to under reporting cannabis use in Canada than in Sweden; however, importantly, most of the data in the current study was collected before the formal legalization of cannabis in Canada in October 2018. Another noteworthy difference is that Gajecki et al. found that baseline problematic alcohol use negatively affected panic disorder outcomes and that hazardous drug use had a negative effect on social anxiety outcomes. In contrast, we found no such differences with changes in social anxiety and panic disorder symptoms.

### 4.4 Limitations

This study has several limitations. First, only self-report data on substance use was collected, which could mean that clients under-report or hide use. Secondly, AUDIT and similar screening instruments tend to render a substantial number of false positives, something that should be taken into consideration when interpreting the data (Stewart and Connors, 2004). Thirdly, we did not collect information on which specific drugs clients were using, and we are thus unable to draw conclusions related to type of drug. It may be that less commonly used drugs (e.g., stimulants) have a negative impact on depression and anxiety outcomes, while a more common drug such as cannabis does not. A fourth limitation is that the sample used in this study does not include clients who have a primary severe problem with alcohol or drugs or individuals at high risk for suicidality as these clients are referred to more targeted or urgent care during the OTU's screening process. Although only a small number of clients were actually excluded due to primary alcohol or drug problems or high suicidality, it remains unknown how well the results of the present study would generalize to those with severe problems with drug use or suicidality. The exclusion of highly suicidal individuals also results in some restriction of range in the data available to analyze related to suicidal ideation and limits our power to detect factors related to changes in suicidal ideation. Fifth, due to the sparse research on substance use among ICBT clients, we decided not to control main outcomes for mass-significance, so as not to miss any potential significant findings. This means that findings may be spurious, and should be interpreted with caution. Sixth, course completion was assessed using website log-in data and it is possible that measures of actual application of skills could also be related to substance use. Seventh, in our sub-analysis, we used LOCF to impute missing data. Although this approach has received some criticism, we selected it as an approach here because missing data were deemed to be not-missing-at-random and in this context LOCF represents a conservative approach as it assumes no further symptom improvement in individuals who leave the program early (Jakobsen et al., 2017). Lastly, since we did not assess alcohol or drug use after treatment or at follow-up, we are unable to comment on whether drug or alcohol use changed over the course of treatment, perhaps due to the treatment itself. A recent study on ICBT for depression found that alcohol consumption had not changed markedly at the 12-month follow-up, despite positive changes in relation to depression (Strid et al., 2019). Future studies could further investigate to what extent ICBT for depression and anxiety affects alcohol and drug use, even when it's not addressed.

### 4.5 Conclusions

A third of clients applying for ICBT had alcohol and/or drug problems. Although those using drugs were less likely to complete treatment, drug use was not associated with depression or anxiety outcomes. Our findings therefore support continued referral of those with comorbid problematic alcohol use and drug problems to ICBT for depression and anxiety when the alcohol and/or drug use is deemed not to be the primary concern. Considering the fact that our results differed from the previously published study (Gajecki et al., 2014), these results need to be replicated in other settings. Although this study's findings suggest that people with comorbid hazardous alcohol use or drug use may continue to be admitted to ICBT programs for anxiety and depression, alcohol/substance use that is not addressed can escalate to more severe forms of use with time, and therefore these conditions remain clinically relevant. Future research should study how alcohol and drug use might be addressed among clients receiving ICBT for depression and anxiety, and whether alcohol and drug problems decrease in response to ICBT. For clients who have comorbid depression/anxiety and alcohol/drug problems it would be instructive in future trials to directly compare ICBT for anxiety/depression and ICBT for alcohol/drug problems on relative efficacy for addressing anxiety/depression and alcohol/drug problems.

## Funding

This research was supported by funding provided by the 10.13039/501100000024Canadian Institutes of Health Research (reference number 152917). Additionally, the Online Therapy Unit is funded by the Saskatchewan Ministry of Health. Funders had no involvement in the study design, collection, analysis, or interpretation of the data. N.T. and B.F.D. are funded by the Australian Government to operate the national MindSpot Clinic.

## Declaration of competing interest

The authors declare that they have no known competing financial interests or personal relationships that appear to have influenced the work reported in this paper.

## Footnotes

1 Item 3 was the most endorsed item among users (94.6%). However, this item was only administered to those already reporting drug use, and as the lowest response alternative in this case indicates “0”, this item was largely redundant with item 1.
